# The stimulatory effect of Thuricin 17, a PGPR-produced bacteriocin, on canola (*Brassica, napus* L.) germination and vegetative growth under stressful temperatures

**DOI:** 10.3389/fpls.2022.1079180

**Published:** 2022-12-23

**Authors:** Mahtab Nazari, Iraj Yaghoubian, Donald L. Smith

**Affiliations:** Department of Plant Science, McGill University, Montreal, QC, Canada

**Keywords:** *Brassica napus*, bacteriocin, stressful temperatures, germination, vegetative growth

## Abstract

Exposure to unfavorable conditions is becoming more frequent for plants due to climate change, posing a threat to global food security. Stressful temperature, as a major environmental factor, adversely affects plant growth and development, and consequently agricultural production. Hence, development of sustainable approaches to assist plants in dealing with environmental challenges is of great importance. Compatible plant-microbe interactions and signal molecules produced within these interactions, such as bacteriocins, could be promising approaches to managing the impacts of abiotic stresses on crops. Although the use of bacteriocins in food preservation is widespread, only a small number of studies have examined their potential in agriculture. Therefore, we studied the effect of three concentrations of Thuricin17 (Th17), a plant growth-promoting rhizobacterial signal molecule produced by *Bacillus thuringiensis*, on germination and vegetative growth of canola (*Brassica napus* L.) under stressful temperatures. Canola responded positively to treatment with the bacterial signal molecule under stressful temperatures. Treatment with 10 ^-9^ M Th17 (Thu2) was found to significantly enhance germination rate, seed vigor index, radical and shoot length and seedling fresh weight under low temperature, and this treatment reduced germination time which would be an asset for higher latitude, short growing season climates. Likewise, Thu2 was able to alleviate the adverse effects of high temperature on germination and seed vigor. Regarding vegetative growth, interestingly, moderate high temperature with the assistance of the compound caused more growth and development than the control conditions. Conversely, low temperature negatively affected plant growth, and Th17 did not help overcome this effect. Specifically, the application of 10 ^-9^ (Thu2) and 10 ^-11^ M (Thu3) Th17 had a stimulatory effect on height, leaf area and biomass accumulation under above-optimal conditions, which could be attributed to modifications of below-ground structures, including root length, root surface, root volume and root diameter, as well as photosynthetic rate. However, no significant effects were observed under optimal conditions for almost all measured variables. Therefore, the signal compound tends to have a stimulatory impact at stressful temperatures but not under optimal conditions. Hence, supplementation with Th17 would have the potential as a plant growth promoter under stressed circumstances.

## Introduction

Germination and vegetative growth are fundamentals of crop production. Rapid, uniform germination and emergence under various climatic conditions play a pivotal role in global food security. Abiotic and biotic factors including extreme temperature, moisture, light, nutrient availability, soil-borne pests and non-pathogen organisms, influence seed germination and establishment (Wang et al., 2016; Lamichhane et al., 2017). Among these, temperature is one of the major environmental factors influencing various plant functions, including seed dormancy and germination. Previous studies have emphasized the role of temperature in the regulatory network of seed germination (Xia et al., 2018; Zhou et al., 2018; Suriyasak et al., 2020; Xue et al., 2021). Temperature controls metabolic and cellular mechanisms, including phytohormone contents, mostly gibberellins and abscisic acid, and determines germination capability and rate (Oracz and Karpiński, 2016; Urbanova and Leubner-Metzger, 2016; Wang et al., 2018b). Some plant seeds germinate across a wide range of temperatures, while others germinate only at a narrow range of temperatures. Canola *(Brassica napus [L.])* has been widely cultivated and is one of the most important oilseeds and biodiesel fuel crops. However, cool spring temperatures slow germination and emergence of spring-sown *B. napus*, which delays flowering in mid-summer. Hence, strategies that improve early germination impact canola yield in cooler (higher latitude and altitude) climates. After seed germination, sufficient vegetative growth is required for the development of roots to uptake water and nutrients, shoots to support aboveground stems, and leaves for photosynthesis and biomass accumulation. However, climate change significantly increases the occurrence of abiotic stresses that adversely affect vegetative growth and development, and ultimately crop production. In such cases, sustainable practices and adopting environmentally friendly approaches can enhance resource efficiency and crop production under stressful conditions. Application of plant-associated microbes, or signal compounds produced by them, such as bacteriocins, can increase plant resistance through direct and indirect mechanisms, including nutrient acquisition, phytohormone production, siderophore production, biocontrol and induced systematic resistance to biotic stressful conditions (Khan et al., 2019; Xia et al., 2020; Antar et al., 2021; Lyu et al., 2021; Shah et al., 2021). Often, plant-microbe interaction signals positively influence plant growth at nearly all stages, from germination to seedling establishment, flowering, seed development and maturity (Gautam et al., 2016; Schwinghamer et al., 2016; Arunachalam et al., 2018). One of the well-known signal molecules is lipo-chitooligosaccharide (LCO) which specifies host-symbiont crosstalk communication and mediates rhizobia-legume associations (Oldroyd et al., 2011; Zipfel and Oldroyd, 2017). In addition, bacteriocins, a specific type of signal molecule with potential positive impacts on agriculture, are yet less known. They can control microbial population dynamics by excretion of versatile compounds known as bacteriocins acting as either a “a never ending arms race” (Riley, 1998) against competitors or, in some cases, signalling compounds are involved in plant colonization and plant growth stimulation. Bacteriocins are antimicrobial peptides produced by bacterial ribosomes and secreted by the bacteria (Arnison et al., 2013). They differ from antibiotics in that they hinder organisms that are closely related to the producer strains, and are active at extremely low concentrations (Mak, 2018). Bacteriocins can sometimes be efficient biocontrol agents in the food and pharmaceutical industries while less attention has been paid to their potential in agriculture. The capacity for bacteriocin production by plant growth-promoting rhizobacteria (PGPR) has been reported in some limited studies, such as bacteriocin exertion by *Pseudomonas fluorescens SF39a*, isolated from the wheat rhizosphere, which suppresses the growth of the phytopathogenic *Pseudomonas* and *Xanthomonas* strains (Godino et al., 2016). Bacteriocins synthesized by rhizobia have been named “Rhizobiocins” (Schwinghamer, 1975). For instance bacteriocin-substances have been isolated by *Bradyrhizobium japonicum* (Gross and Vidaver, 1978; Mojgani, 2017) and *Rhizobium leguminosarum* related strains (Hirsch, 1979; Hafeez et al., 2005). Among rhizosphere microbiome, *Bacillus* species were one of the first groups studied for production of diverse bacteriocins. So far, there have been reports of the synthesis of 18 bacteriocins from *B. thuringiensis* (Mojgani, 2017). Yet, bacteriocin excretion by *Bacillus* PGPR is poorly understood, and none have been examined as thoroughly as Th17 for plant growth promotion; Th17 was discovered in our laboratory and is produced by *B. thuringiensis* non-*Bradyrhizobium* Endophytic Bacterium 17 (BtNEB17), an endophytic bacterium isolated from soybean root nodules [patent by Smith et al. (2008)]. Th17 is highly resistant to heat, stable across a pH range of 1.0–9.25 and is a low molecular weight peptide of 3.162 kDa with anti-microbial activity and plant growth promotion ability, particularly under stressful conditions (Jung et al., 2011; Prudent et al., 2015; Schwinghamer et al., 2015; Subramanian et al., 2016; Schwinghamer et al., 2016; Nazari and Smith, 2020). Interestingly, this bacteriocin could alter physiological and biochemical attributes of plants including increases in photosynthetic rate and antioxidant enzymes contents of plants under normal and stressed conditions (He, 2009; Jung et al., 2011) We should note that more recent characterization of Th17 has shown sufficient differences from our original understanding of its composition that we are considering renaming it to Bacillin 20. Although studies on the efficacy of plant-microbe interactions are abundant, there is a lack of studies around the agricultural potential of bacteriocins. We, therefore, conducted a series of experiments to examine the stimulatory effect of Th17, a plant growth promoting rhizobacteria signal molecule, on germination and vegetative growth of canola under contrasting temperature conditions. The aim is to develop an environmentally friendly approach to stimulation of plant growth and development in a constantly changing environment.

## Materials and methods

### Isolation and purification of Th17

BtNEB17 was grown in King’s medium B as previously described (Gray et al., 2006). Cells for the initial broth inoculum were taken from plated material and cultivated in 250 mL flasks with 50 mL medium. For 48 h, the bacterium was cultivated at 28 ± 2 °C on an orbital shaker rotating at 150 rev min ^-1^. For the initial culture, 5 mL of subculture was inoculated into 2000 mL of broth, and the culture was grown under the same conditions. Bacterial populations were assessed by an Ultrospec 4050 Pro UV/Visible Spectrophotometer at 600 nm 96 h after initiation. A cell-free supernatant (CFS) containing BtNEB17 was obtained through centrifuging the bacterial culture at 13,000 g for 10 min, followed by analytical-HPLC identification. For partial purification, 800 mL of butanol was added to 2000 mL of bacterial culture for 12 h then the collected upper layer was extracted under vacuum in rotary evaporator. The viscose extract was collected with 12 mL of 30% Acetonitrile and centrifuged at 13,000 g for 13 min, and the supernatant was collected for chromatography (Gray et al., 2006). Th17 was selected based on peaks and retention times. After being lyophilized, the samples were kept at 20 °C for dilution to the required concentrations. For all experiments, 10^-7^ (Thu1), 10^-9^ (Thu2) and 10^-11^ (Thu3) M of Th17 were prepared based on the molecular weight of Th17.

### Germination experiments

Canola seeds (cultivar L233P) were disinfected with 20% sodium hypochlorite and rinsed with distilled water before applying treatments. Twenty-five sterilized seeds were placed on petri plates (100 x 15 mm) lined with filter paper (QualitativeP8). The plates were randomly treated with 10 mL of Thu1, Thu2, Thu3 solution and distilled water. Petri dishes were then sealed with parafilm to prevent water loss and arranged in a completely randomized design, with four replications for each treatment, in growth chambers. Growth chambers were set to low (5 °C), optimal (20 °C), high (30 °C) temperatures, 70% humidity, and zero illumination. The total number of germinated seeds were counted at 24 h intervals, for 72 h at optimum and high temperature and 7 days for low temperature. Radical and shoot length were measured for all experiments after 7 days, and seedlings were weighted. Each experiment was repeated twice, and the data was pooled for analysis.

### Plant growth experiments

**Figure 1:**
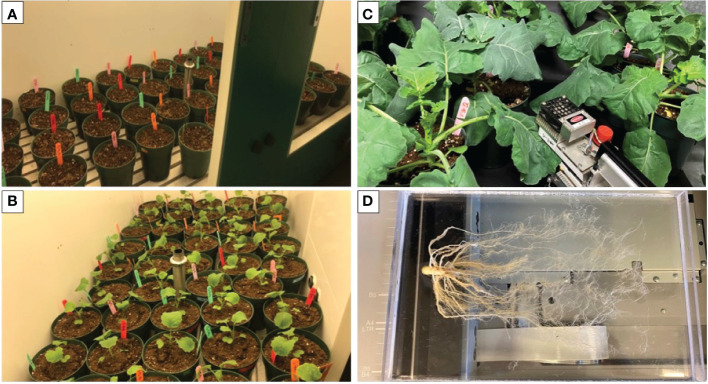
**(A)** Canola pots after seeding in the growth chamber (22/18 °C), **(B)** 3-week-old canola plants grown under optimal conditions (before starting temperature stress), **(C)** Physiological measurements with Licor from 7-week-old canola plants, **(D)** 7-week-old canola roots after removing soil particles.

**Figure 2:**
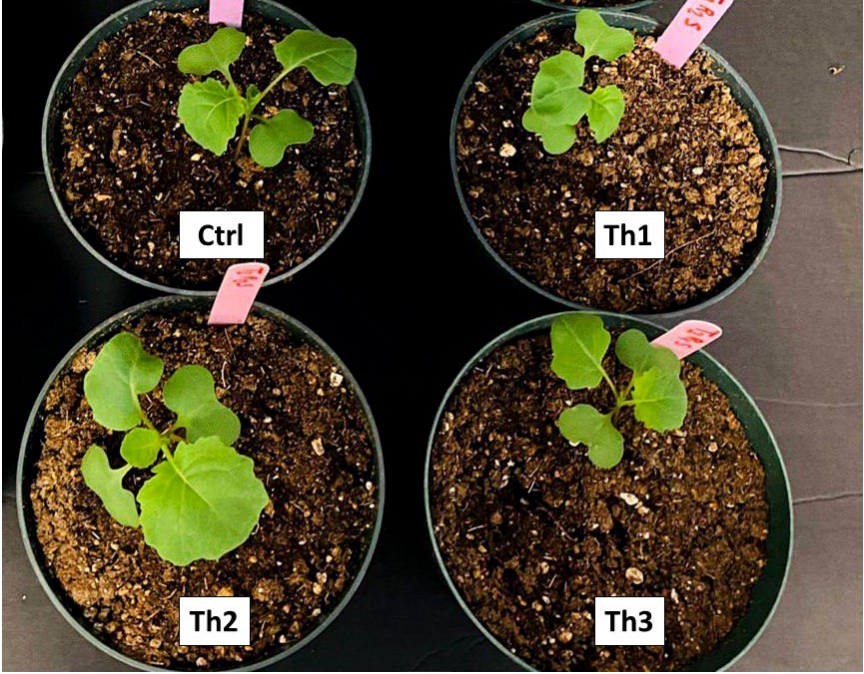
3-week-old canola plants grown under optimal conditions. Ctrl: seeds treated with distilled water, Th1: seeds treated with 10 ^-7^ M Th17, Th2: seeds treated with 10 ^-9^ M Th17, Th3: seeds treated with 10 ^-11^ M Th17.

Th17 was applied as a pre-planting seed treatment, seeds were soaked in 10 mL Thu1, Thu2, Thu3 solution and distilled water, and root irrigation after the stress induction time. Canola seeds (5 seeds per pot) were placed in 10 cm pots filled with AGRO MIX^®^ G10 media, which was a mix of peat, limestone, balanced nutrient materials, with micronutrients, gypsum and wetting agent. This growth medium was chosen based on root application of Th17 and the ability of peat to gradually release water and/or the compound. The pots were placed in a growth chamber (Conviron R, Canada) at 22/18 °C day/night, with a photoperiod of 14/10 h light/darkness cycle, 60-70% relative humidity and photosynthetic irradiance of 350–400 µmol m^−2^ s ^−1^ (**Figure 1A**). After a week, the seedlings were counted, and the plants were thinned to one seedling per pot. Plants were regularly watered with half strength Hoagland’s solution and grown in the growth chamber until the end of the third week (**Figure 1B**). Then, 3-week-old seedlings (**Figure 2**) were randomly selected to transfer to the low (10/5 °C), optimum (22/18 °C) and high temperature (30/25 °C) chambers to determine the effect of stressful temperatures on the vegetative growth of canola. Plants were watered semi-weekly with 250 mL of either water (for controls) or Th17 (for Th17-treated plants). Following the start of the temperature treatment, the plants in each case were allowed to develop for 4 weeks before being sampled for data collection. A LI-COR 6400 portable photosynthesis metre (LI-COR Lincoln, NE), was used to measure the photosynthetic rate, transpiration rate, stomatal conductance and CO_2_ concentration inside the leaves. Readings were taken on a weekly basis (4, 5, 6, 7 weeks) after the 3-week-old seedling stage. An upper-most fully expanded leaf was used for determination of physiological variables between 10:00 and 14:00 h (**Figure 1C**). Plants were sampled at the end of the experiment for the following variables: plant height, leaf area, fresh weight, dry weight and root variables. After harvesting plants and measuring morphological traits, roots were coarsely shaken off to remove soil (destructive methods). Roots were then washed with water and adhering soil particles removed with brushes (**Figure 1D**). Total roots were scanned using EPSON-Expression 11000XL then root length, root volume, total root surface and root diameter were analyzed by WinRHIZO^TM^ Pro software. Each experiment described above was repeated twice with four replicates, and data were pooled for analysis.

### Data analysis

**Figure 3:**
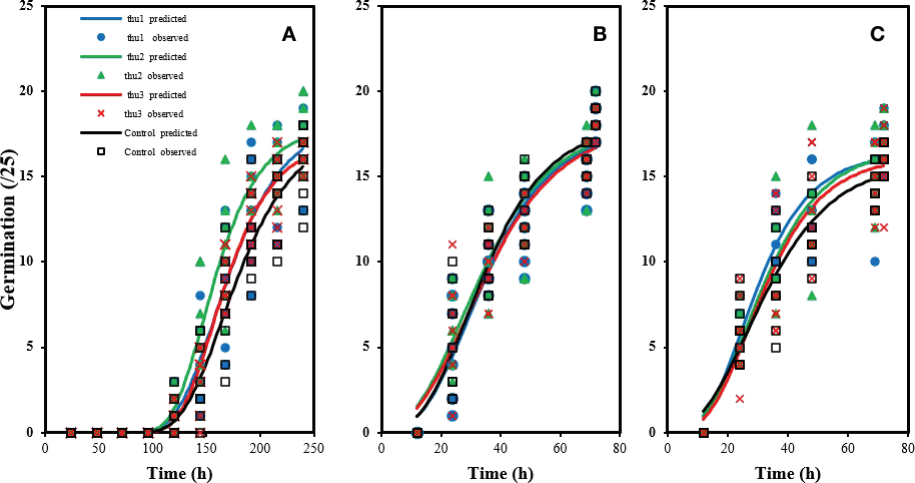
Effects of temperature and Th17 concentrations on germination rate at 5 °C **(A)**, 20 °C **(B)**, 30 °C **(C)**. thu1: 10 ^-7^ M Th17, thu2: 10 ^-9^ M Th17, thu3: 10^-11^ M Th17. Points and lines represent observed and predicted values, respectively.

Data from the experiments were analyzed, based on a completely randomized factorial design, using the SAS Statistical Package 9.3 (SAS Institute Inc., Cary, NC, USA). For germination data, observed values were analysed similarly to other variables and a non-linear regression model was fitted to predict the probability of germination, in **Figure 3** (Piegorsch and Bailer, 2005; Schwinghamer et al., 2015). Duncan’s multiple comparison test was used when there was a significant difference at the 95% confidence level.

## Results

### Germination

There were no significant differences in germination rate under optimal conditions (20 °C) (**Figure 3B**). The control had the highest germination rate, which did not differ significantly from Th17-treated seeds. The lowest germination rate under optimal conditions was for seeds treated with the lowest concentration, Thu3. With increasing temperature to 30 °C **Figure 3C**, germination decreased although applying Th17 helped alleviate the negative effect of high temperature on germination; in particular Thu2 treatment increased germination significantly. Cold stress strongly affected germination (**Figure 3A**); about 100 h were required to start germination, and it increased slowly after that. In marked contrast, the first germination was recorded approximately 12 h after onset of the experiment at 20 °C. Under cold stress conditions, the interaction of temperature and the compound application was significant (*p* < 0.002), and application of Th17 significantly influenced germination, mainly mid-range concentration (Thu2) maximized germination rate.

#### Germination time

**Figure 4:**
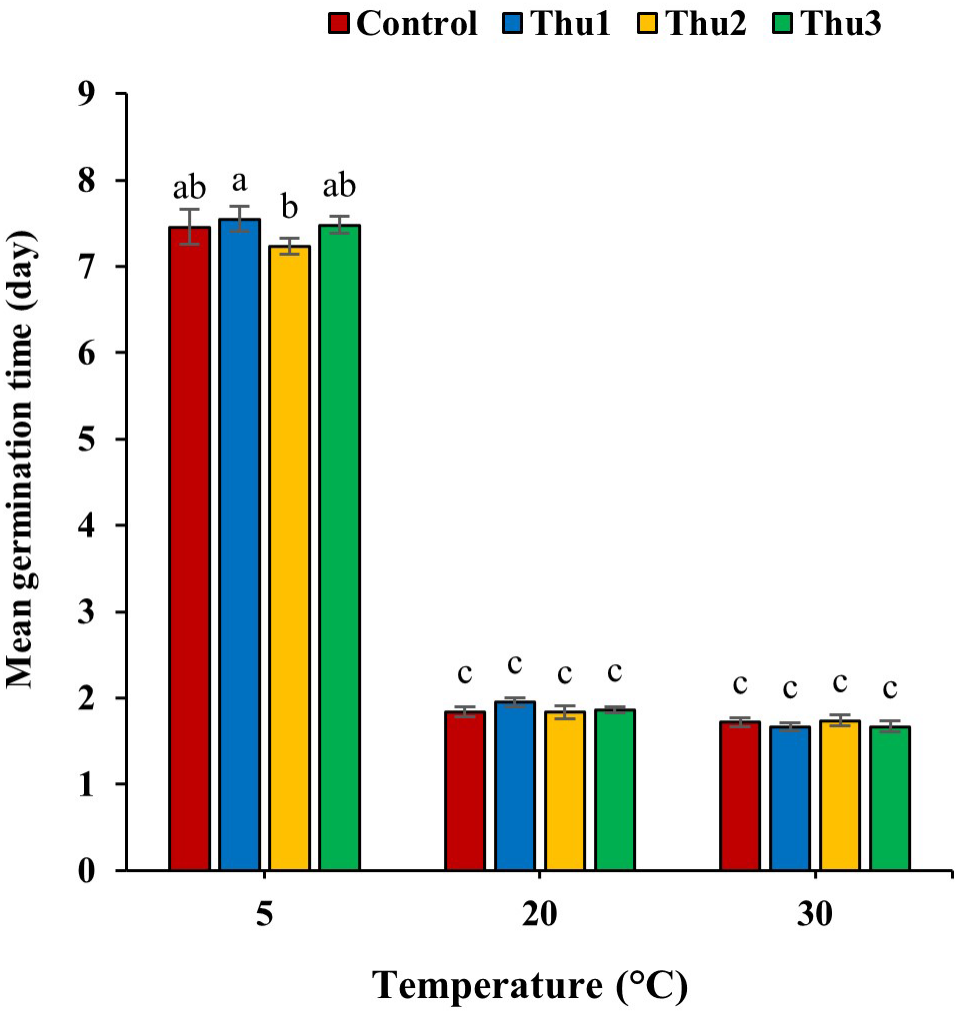
Effects of temperature and Th17 concentrations on mean germination time. Thu1: 10 ^-7^ M Th17, Thu2: 10 ^-9^ M Th17, Thu3: 10^-11^ M Th17. Each bar represents mean ± standard error (n=8). Means with the same letters are not significantly different (*p* < 0.05).

Germination time, one of the main indicators of germination process, was altered by Th17 application and temperature (**Figure 4**). In general, temperature caused a decrease or increase in germination time whereas the application of different Th17 levels minimized germination time under low temperature, except for Thu1, which showed a maximum time of 7.54 days. Thu2 treatment caused the shortest germination time at 5 °C, at 7.23 days while this time was 1.84 and 1.66 days for control and Thu1 at 20 and 30 °C, respectively.

#### Length of radical and shoot

**Figure 5:**
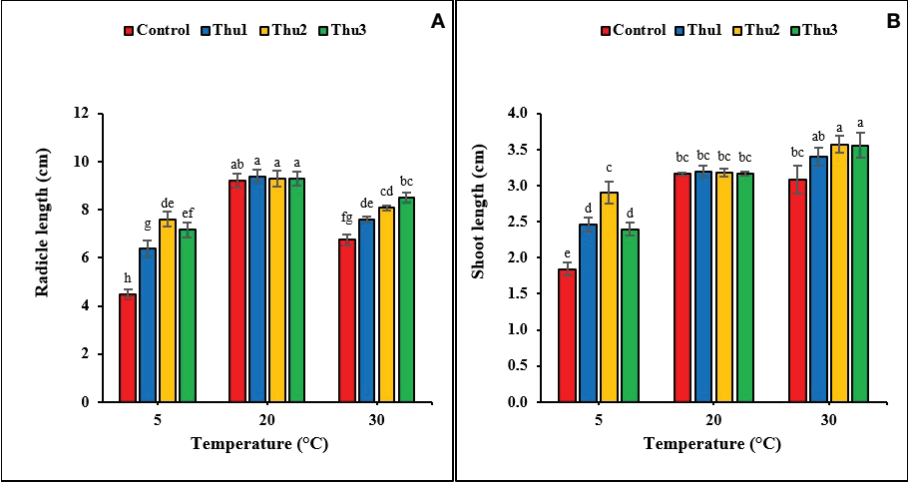
Effects of temperature and Th17 concentrations on radical **(A)** and shoot length **(B)**. Thu1: 10 ^-7^ M Th17, Thu2: 10 ^-9^ M Th17, Thu3: 10^-11^ M Th17. Each bar represents mean ± standard error (n=8). Means with the same letters are not significantly different (*p* < 0.05).

It is clear from data that radical and shoot length significantly responded to the interaction of Th17 and temperatures (*p* < 0.001 and *p* < 0.002, respectively) (**Figure 5**). The greatest radical length was for Thu1 treatment, 9.38 cm, at optimal temperature; however, it was not significantly different from the control. Although increasing the temperature reduced the radical length, using Th17 at low concentrations (Thu3) increased length and reduced the harmful effects of high temperature. Under low temperature, the longest and shortest radical were observed for Thu2 treated seedlings (7.6 cm) and the control (4.5 cm), respectively (**Figure 5A**). For shoot length, the tallest shoots (3.6 cm) were from Thu2 treated plants at 30 °C, which were about 0.5 cm longer than the control (**Figure 5B**). Likewise, shoot length increased at low temperature from 1.84 for the control (the shortest across all temperatures) to 2.9 cm for Thu2 seedlings. Surprisingly, Th17 application could not significantly affect shoot length compared to untreated seeds under optimal temperature conditions.

#### Seedling weight and length

The interaction of temperature and Th17 concentrations on radical (*p* < 0.001), shoot (*p* < 0.001) and seedling weight (*p* < 0.001) was significant. Cold stress caused a sharp decrease in the fresh weight of radicals, shoots and seedlings; however, Th17 enhanced seedling fresh weight, from 0.54 mg for control to 0.66 mg for Thu2. Conversely, under optimal conditions, radical, shoot and seedlings of untreated seeds were heavier, 0.38, 0.64 and 1.02 g, respectively, than Th17 treatments. At high temperature, similar to the optimum temperature, the highest fresh weight of radical and shoot was for control seeds. In addition, there were no significant effects of treatment on seedling length at 20 °C; however, the greatest level was for Thu1 treated seedlings (12.58 cm); the shortest seedling was that of the control under low temperature conditions (**Table 1**).

**Table 1:** Effects of temperature and Th17 concentrations on radicle, shoot and seedling fresh weight and length.

Temperature (°C)	Th17 Treatments	Radicle fresh weight (g)	Shoot fresh weight (g)	Seedling fresh weight (g)	Seedling length (cm)
**5**	**Control**	0.17 ± 0.03 e	0.36 ± 0.02 e	0.54 ± 0.04 d	6.33 ± 0.57 g
	**Thu1**	0.19 ± 0.05 de	0.40 ± 0.04 e	0.60 ± 0.8 cd	8.85 ± 1.15 f
	**Thu2**	0.21 ± 0.04 de	0.45 ± 0.02 de	0.66 ± 0.05 c	10.5 ± 1.34 cd
	**Thu3**	0.18 ± 0.02 e	0.38 ± 0.04 e	0.57 ± 0.05 cd	9.56 ± 1.11 ef
**20**	**Control**	0.38 ± 0.09 a	0.64 ± 0.11 ab	1.02 ± 0.12 a	12.37 ± 0.97 a
	**Thu1**	0.28 ± 0.06 b	0.53 ± 0.06 cd	0.81 ± 0.08 b	12.58 ± 0.85 a
	**Thu2**	0.28 ± 0.05 b	0.50 ± 0.06 cd	0.78 ± 0.06 b	12.47 ± 1.12 a
	**Thu3**	0.24 ± 0.03 bcd	0.51 ± 0.05 cd	0.75 ± 0.06 b	12.47 ± 0.85 a
**30**	**Control**	0.27 ± 0.06 bc	0.69 ± 0.2 a	0.63 ± 0.18 cd	9.82 ± 1.12 de
	**Thu1**	0.23 ± 0.05 cde	0.57 ± 0.11 bc	0.79 ± 0.14 b	10.99 ± 0.53 bc
	**Thu2**	0.24 ± 0.05 bcd	0.56 ± 0.06 c	0.80 ± 0.08 b	11.65 ± 0.49 ab
	**Thu3**	0.22 ± 0.03 cde	0.57 ± 0.09 bc	0.79 ± 0.11 b	12.06 ± 0.96 a

Thu1: 10 ^-7^ M Th17, Thu2: 10 ^-9^ M Th17, Thu3: 10^-11^ M Th17. Each value represents mean ± standard error (n=8). Means with the same letters are not significantly different (*p* < 0.05).

#### Seed vigor

**Figure 6:**
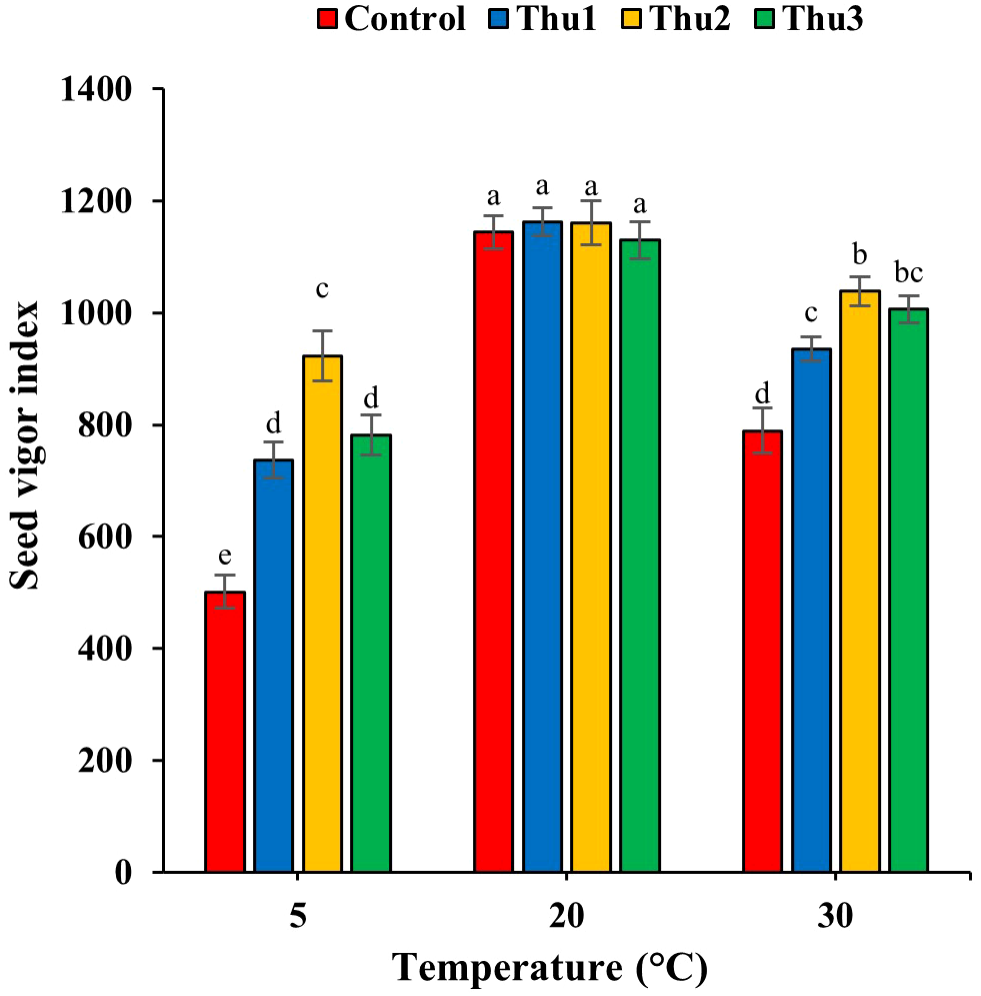
Effects of temperature and Th17 concentrations on seed vigor index. Thu1: 10 ^-7^ M Th17, Thu2: 10 ^-9^ M Th17, Thu3: 10^-11^ M Th17. Each bar represents mean ± standard error (n=8). Means with the same letters are not significantly different (*p* < 0.05).

Data in **Figure 6** reveals the adverse effects of high and low temperatures on seed vigor; Th17 application significantly mitigated detrimental impacts of stressful temperatures (*p* < 0.001) while seed vigor decreased significantly by 33.99 and 56.18% for control seeds in response to high and low temperatures, respectively.

### Plant growth and development

#### Plant height

**Figure 7:**
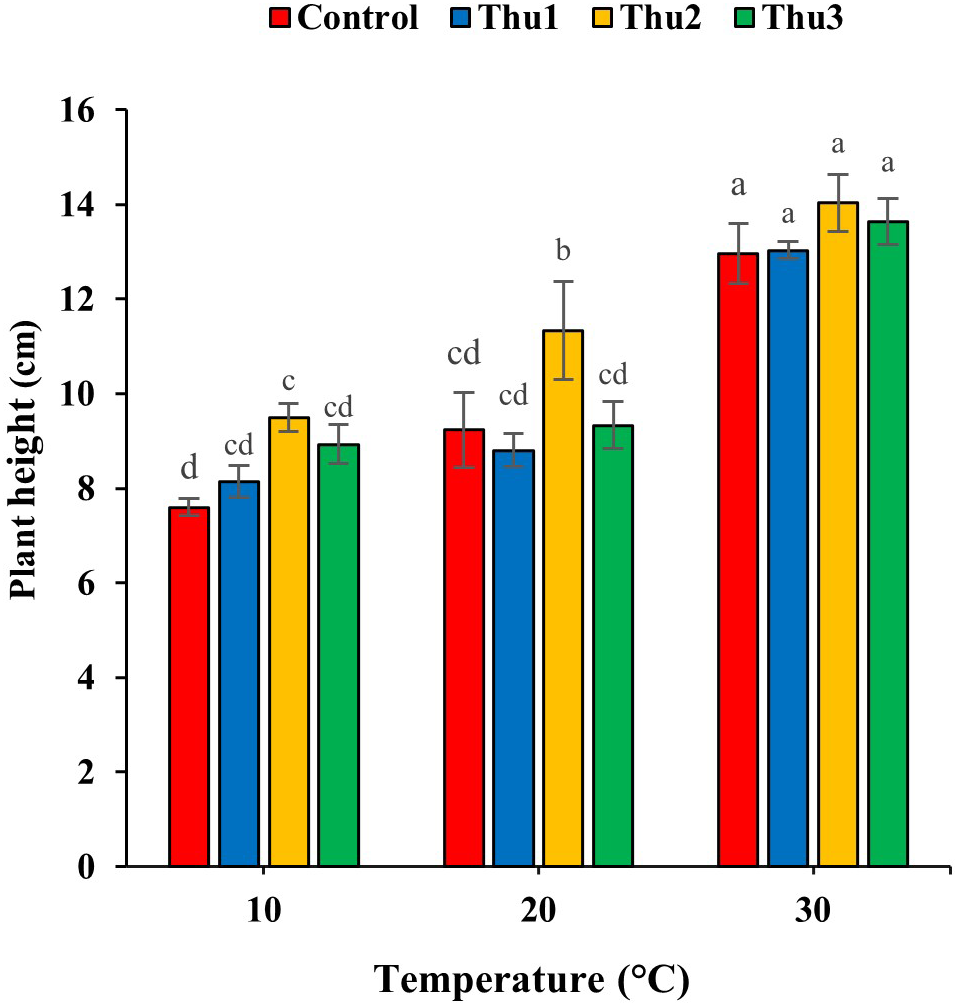
Effects of temperature and Th17 concentrations on canola height. Thu1: 10 ^-7^ M Th17, Thu2: 10 ^-9^ M Th17, Thu3: 10^-11^ M Th17. Each bar represents mean ± standard error (n=8). Means with the same letters are not significantly different (*p* < 0.05).

Treating seeds with Th17 increased plant height, specifically Thu2 (**Figure 7**); the compound enhanced height by 25, 22.75 and 8.25% over control, at low, optimal and high temperatures, respectively.

#### Leaf area

**Figure 8:**
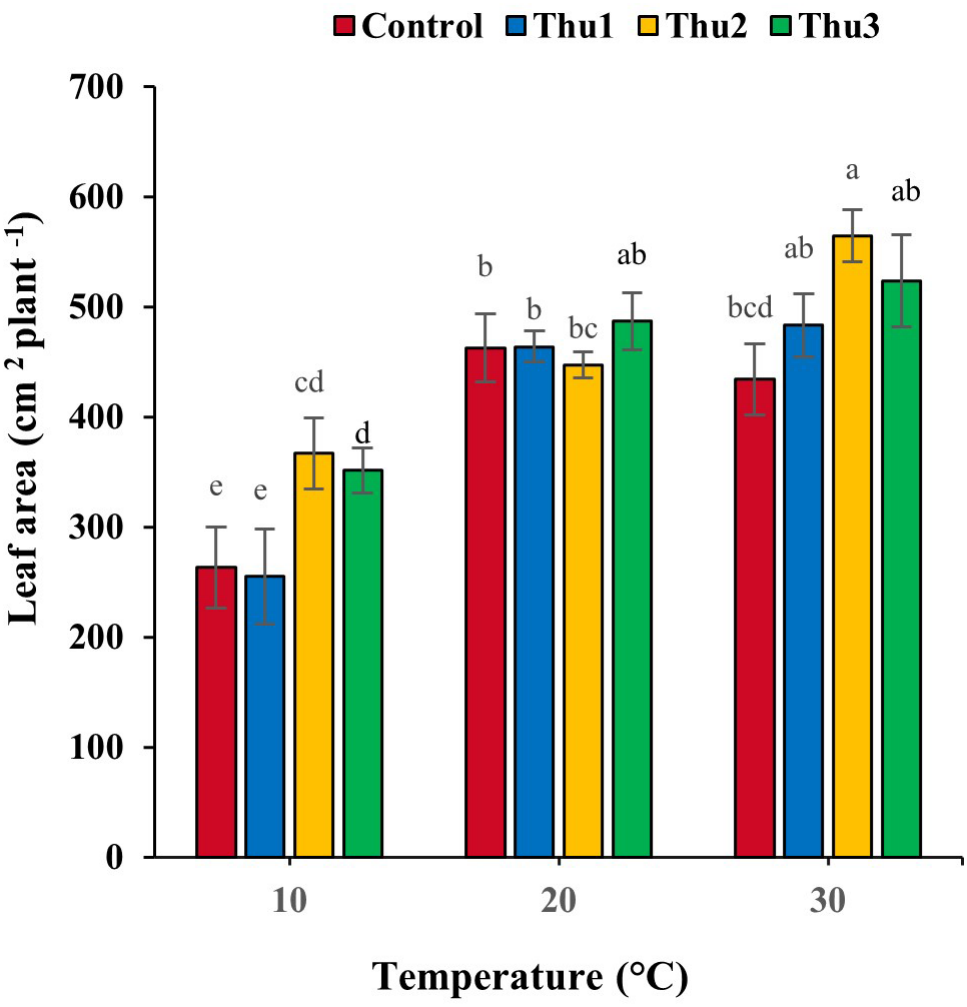
Effects of temperature and Th17 concentrations on canola leaf area. Thu1: 10 ^-7^ M Th17, Thu2: 10 ^-9^ M Th17, Thu3: 10^-11^ M Th17. Each bar represents mean ± standard error (n=8). Means with the same letters are not significantly different (*p* < 0.05).

Leaf area responded strongly to Th17 at stressful temperatures (**Figure 8**). It decreased by 6.19 and 43.11% for the controls at high and low temperatures, respectively. Under optimal conditions, no stimulatory effect of Th17 was observed, except for Thu3, whereas positive responses occurred at stressful temperatures, particularly for Thu2 plants where leaf area increased by 39 and 30%, at 10 and 30 °C temperatures, respectively, compared to the control.

#### Biomass

**Figure 9:**
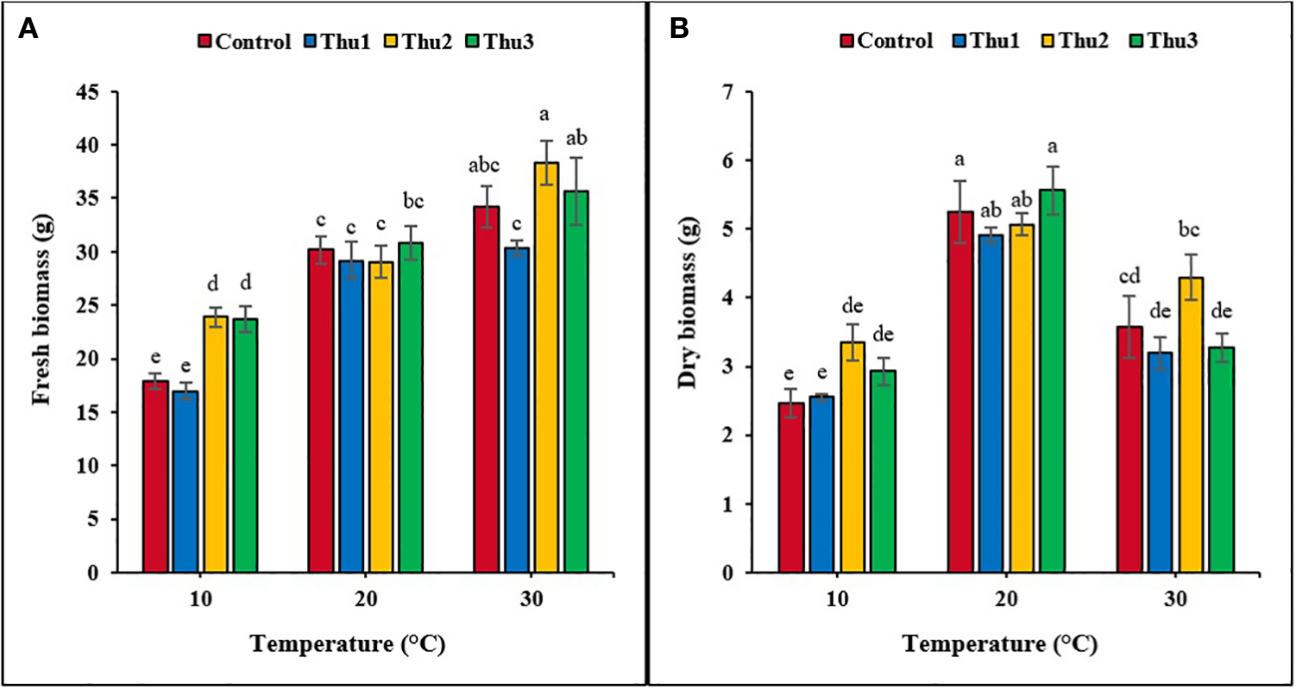
Effects of temperature and Th17 concentrations on canola fresh **(A)** and dry **(B)** biomass. Thu1: 10 ^-7^ M Th17, Thu2: 10 ^-9^ M Th17, Thu3: 10^-11^ M Th17. Each bar represents mean ± standard error (n=8). Means with the same letters are not significantly different (*p* < 0.05).

**Figure 10:**
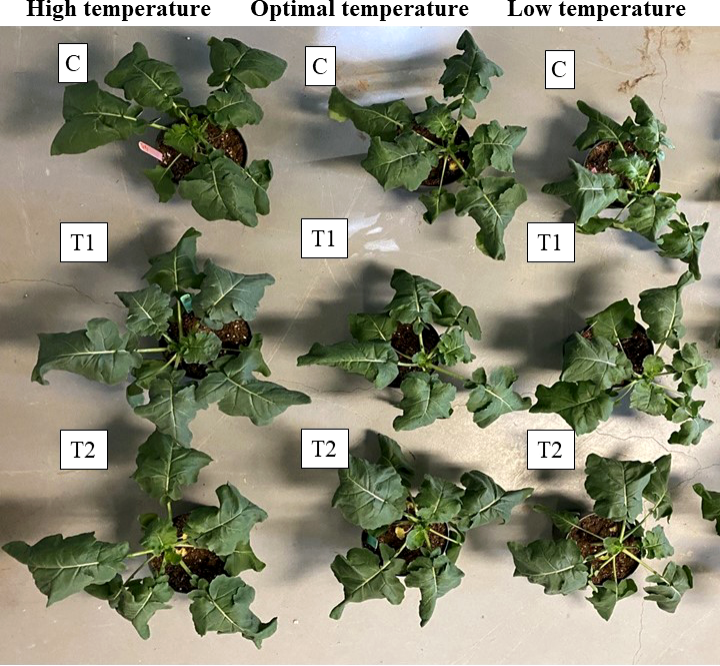
The effect of different temperatures and two of the best Th17 concentrations on canola growth and development. 7-week-old canola plants grown under high (30/25 °C, the first column of plants), optimal (22/18 °C, the second column of plants) and low (10/5 °C, the third column of plants) temperatures. C: seeds treated with distilled water and plants watered with water during temperature stress, T1: 10^-9^ M Th17 applied as seed treatment before sowing and root irrigation during temperature stress induction, T2: 10^-11^ M Th17 applied as seed treatment before sowing and root irrigation during temperature stress induction.

Plants grown at 30 °C had the highest fresh biomass accumulation (**Figures 9**, **10**), particularly those treated with Thu2, with 38.33 g. Conversely, much less biomass accumulated under low temperature conditions although it increased by 25% when plants treated with Thu2 (**Figure 9A**). Our data clearly demonstrated that dry biomass significantly declined under stressful temperatures; however, Th17 application showed positive effects, with dry biomass increasing by 20.16 and 43.49% for Thu2 compared to the control at 30 and 10 °C, respectively. Similarly, dry biomass accumulation increased from 5.24 g for control to 5.56 g for Thu3 under optimal conditions (**Figure 9B**).

#### Root variables

**Figure 11:**
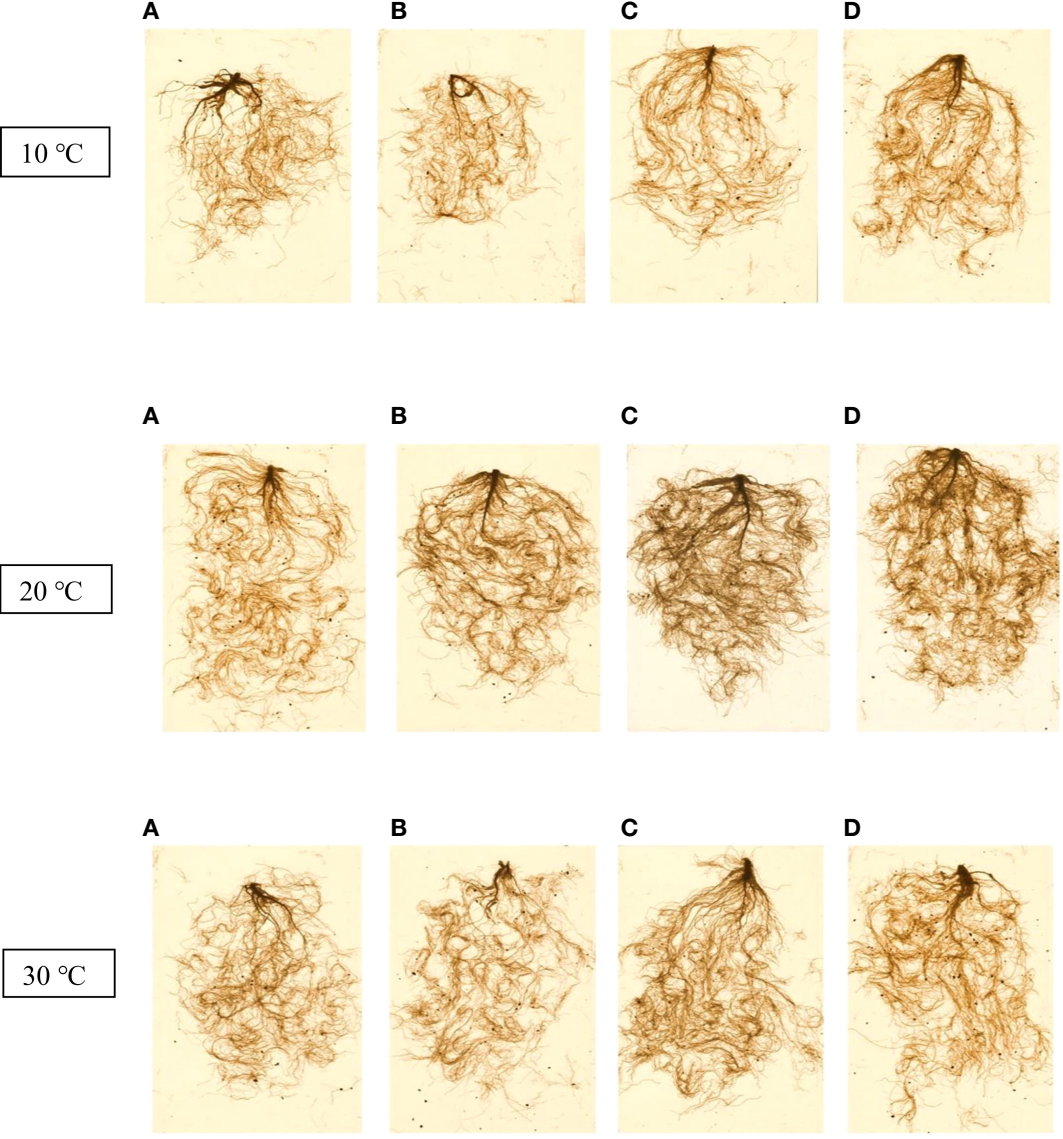
7-week-old canola root images for 10, 20 and 30 °C, respectively. **(A)** Control, **(B)** Thu1 (10 ^-7^ M Th17), **(C)** Thu2 (10 ^-9^ M Th17), **(D)**: Thu3 (10^-11^ M Th17).

**Table 2** presents the root dry weight, length and root surface data and shows that they were negatively affected by stressful temperatures. Root dry weight ranged from 0.31 g for the control at 10 °C to 0.74 g for Thu3 under optimal conditions. Watering plants with Thu3 enhanced root length and surface across all temperatures (**Figure 11**). Conversely, the highest values for root diameter and volume were detected from Thu2 and Thu3 plants, respectively, at 30 °C.

**Table 2:** Effects of temperature and Th17 concentrations on canola root variables.

Temperature °C	Treatment	Root length (cm)	Root surface (cm^2^)	Root diameter (mm)	Root volume (cm^3^)	Root dry weight (g)
**10**	**Control**	2077.1 ± 193.2 de	220.9± 46.2 c	0.21 ± 0.01 de	1.17 ± 0.12 c	0.31 ± 0.06 g
	**Thu1**	1841.1 ± 447.2 e	228.5 ± 32.7 bc	0.20 ± 0.02 e	1.01 ± 0.16 c	0.30 ± 0.08 g
	**Thu2**	2352.8 ± 99.8 de	258.5 ± 09.8 abc	0.25 ± 0.01 abc	1.27 ± 0.19 c	0.42 ± 0.04 f
	**Thu3**	3139.3 ± 67.2 bc	278.2 ± 27.6 abc	0.24 ± 0.01 cde	1.77 ± 0.36 ab	0.46 ± 0.04 ef
**20**	**Control**	3640.2 ± 563.8 abc	307.7 ± 62.8 ab	0.26 ± 0.02 abc	2.06 ± 0.30 a	0.66 ± 0.13 abc
	**Thu1**	3304.8 ± 213.7 abc	278.0 ± 60.6 abc	0.24 ± 0.04 cde	1.84 ± 0.21 ab	0.64 ± 0.03 bcd
	**Thu2**	3945.4 ± 861.2 ab	303.5 ± 37.1 abc	0.25 ± 0.02 bcd	1.89 ± 0.32 ab	0.70 ± 0.07 a
	**Thu3**	4178.5 ± 688.2 a	329.3 ± 22.3 a	0.25 ± 0.03 bcd	2.06 ± 0.17 a	0.74 ± 0.04 a
**30**	**Control**	2875.8 ± 543.7 cd	247.9 ± 42.5 abc	0.27 ± 0.01 ab	2.08 ± 0.11 a	0.55 ± 0.06 cde
	**Thu1**	2781.6 ± 618.7 cd	251.4 ± 52.6 abc	0.28 ± 0.01 abc	1.92 ± 0.19 ab	0.56 ± 0.06 cde
	**Thu2**	3552.8 ± 99.7 abc	254.9 ± 54.1 abc	0.29 ± 0.01 a	1.95 ± 0.18 ab	0.53 ± 0.02 def
	**Thu3**	3620.2 ± 456.7 abc	306.7 ± 34.4 ab	0.28 ± 0.02 ab	2.10 ± 0.14 a	0.68 ± 0.01 ab

Thu1: 10 ^-7^ M Th17, Thu2: 10 ^-9^ M Th17, Thu3: 10^-11^ M Th17. Each value represents mean ± standard error (n=8). Means with the same letters are not significantly different (*p* < 0.05).

#### Physiological variables

**Figure 12:**
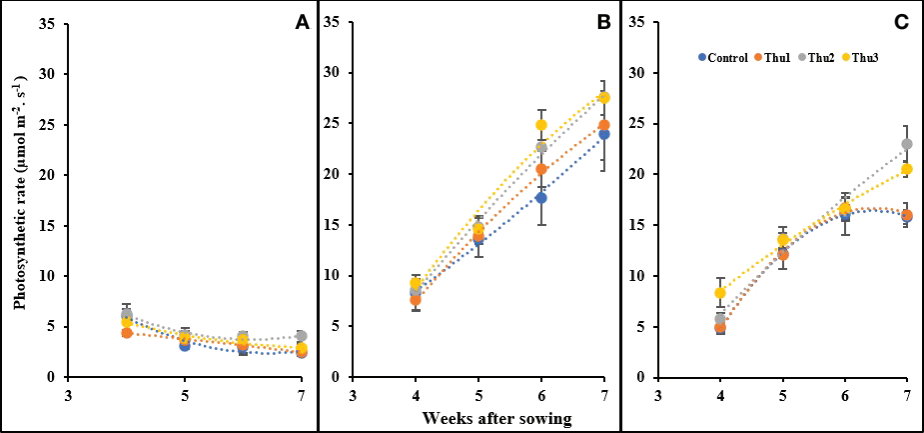
Changes in the photosynthetic rate under application of Th17 at low **(A)**, optimum **(B)** and high **(C)** temperature at 4, 5, 6 and 7 weeks after sowing. Thu1: 10 ^-7^ M Th17, Thu2: 10 ^-9^ M Th17, Thu3: 10^-11^ M Th17. Each point represents mean ± standard error (n=8).

Photosynthetic rate was affected by temperature and Th17 as indicated in **Figure 12**; changes either below or above optimal temperature led to photosynthesis decline. As we expected, plants grown in a cold growth chamber manifested a steady decrease over time, and maximum photosynthesis was observed from Thu2-treated plants in the first week of measurement. Conversely, all treatments had an upward trend under optimal conditions during measurement times, and followed the same general pattern. The trend for 30 °C was increasing until the third measurement then control and Thu1 showed a decrease whereas Thu2 and Thu3 continued to increase; photosynthesis of Thu2 plants was approximately 40% higher than the control in the final measurement.

#### Stomatal conductance

**Figure 13:**
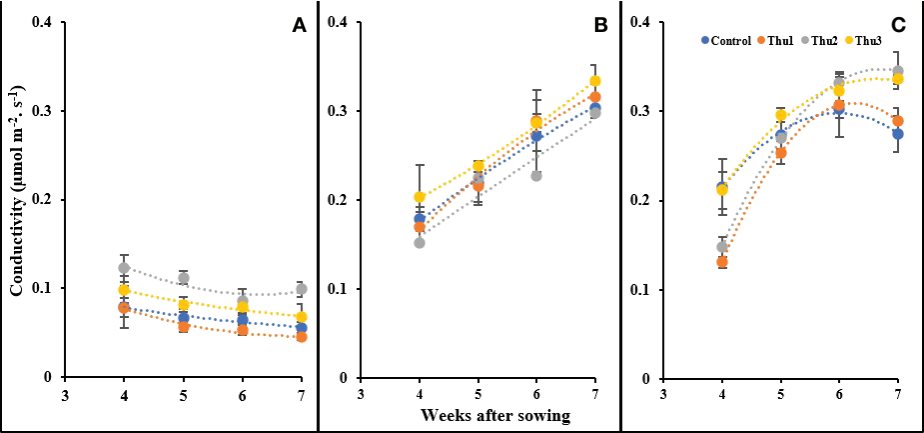
Changes in stomatal conductivity under application of Th17 at low **(A)**, optimum **(B)** and high **(C)** temperature at 4, 5, 6 and 7 weeks after sowing. Thu1: 10 ^-7^ M Th17, Thu2: 10 ^-9^ M Th17, Thu3: 10^-11^ M Th17. Each point represents mean ± standard error (n=8).

As indicated in **Figure 13**, stomatal conductance responded strongly to temperature and concentration of Th17. With rising temperature, conductivity increased; Thu2 treated plants indicated a sharp increase and had the highest conductivity, at 0.345 mol m^-2^ s^-1^, as opposed to control with a gradual downward trend at 30 °C. Under cold temperature, conductivity drastically dropped for all plants; Thu2 plants could maintain their highest capacity for gas transport through the stomata. In clear contrast, a consistent enhancement occurred under optimal conditions, and this upward trend was similar for all treatments.

#### Intercellular CO_2_

**Figure 14:**
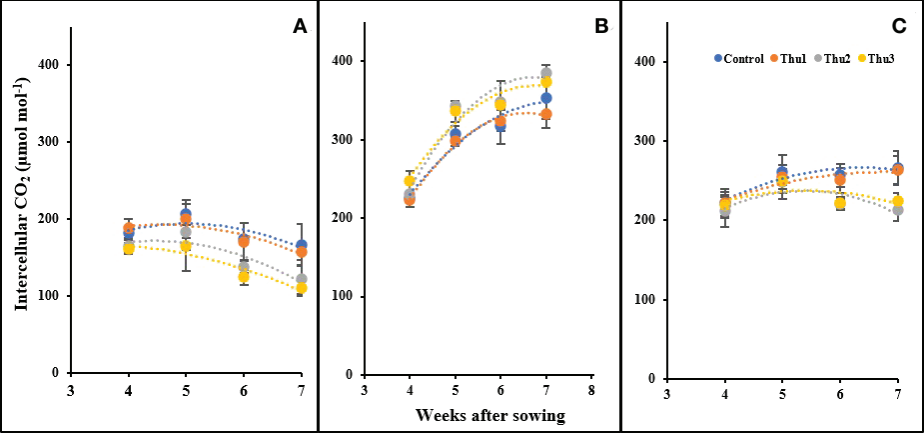
Changes in the intercellular CO_2_ under application of Th17 at low **(A)**, optimum **(B)** and high **(C)** temperature at 4, 5, 6 and 7 weeks after sowing. Thu1: 10 ^-7^ M Th17, Thu2: 10 ^-9^ M Th17, Thu3: 10^-11^ M Th17. Each point represents mean ± standard error (n=8).

There were significant changes in intercellular CO_2_ in response to temperature and Th17 levels (**Figure 14**). Our measurements indicated that the highest levels of intercellular CO_2_ occurred at 20 °C, ranging from 225 µmol mol^-1^ for the control at the first measurement, to 380 µmol mol^-1^ for Thu2 at the last reading. Stressful temperatures both had a downward trend in which control plants absorbed more CO_2_ than treated ones.

#### Transpiration

**Figure 15:**
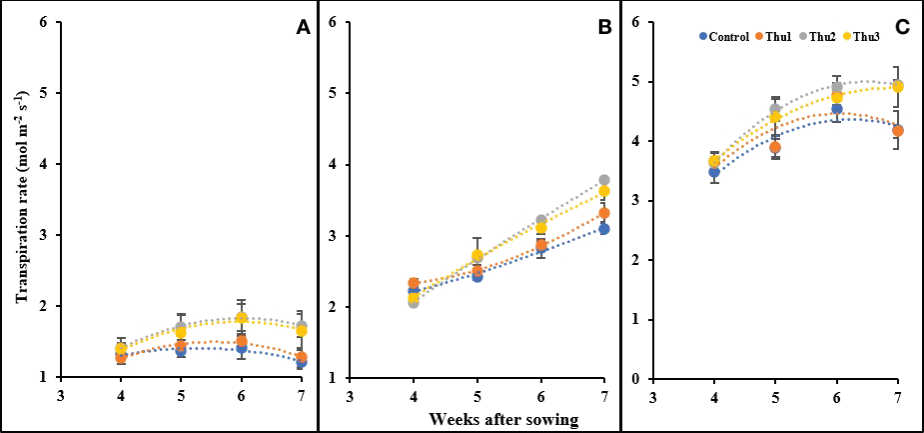
Changes in the transpiration rate under application of Th17 at low **(A)**, optimum **(B)** and high **(C)** temperature at 4, 5, 6 and 7 weeks after sowing. Thu1: 10 ^-7^ M Th17, Thu2: 10 ^-9^ M Th17, Thu3: 10^-11^ M Th17. Each point represents mean ± standard error (n=8).

Transpiration rate was enhanced by increasing temperature; control plants grown at 10 °C had the lowest transpiration (1.21 mol m^-2^ s^-1^) whereas the highest level was for Thu2 treatment (4.95 mol m^-2^ s^-1^) at 30 °C. Interestingly, plants under high stress conditions showed an increase until 6 weeks after sowing, after which transpiration changed only slightly and/or decreased. Conversely, plants maintained a constant upward transpiration over time at optimal temperature. Predictably, at 10 °C, the transpiration rate was very low and slightly changed; Thu2 and Thu3 caused a marginal increase while transpiration for Thu1 and the control treatments showed a negligible decrease **Figure 15**).

## Discussion

### Germination

Seed germination is a very important stage in the life cycle of a plant and one that is very sensitive to both the intrinsic and extrinsic factors (Rifna et al., 2019). Among them, temperature is a major environmental factor determining seed germination (Finch‐Savage and Leubner‐Metzger, 2006). Early germination is an essential element that impacts the production of canola (*B. napus* [L.]). The optimal temperature for canola germination is 22°C (Nykiforuk and Johnson‐Flanagan, 1994; Tasseva et al., 2004). Any temperature either below or above optimal induces uneven germination and, in the field, poor stand establishment, restricting the crop potential to produce yield. In this study, we found that germination and seedling growth indices were negatively affected by both low and high temperatures. Germination was delayed under low temperature conditions, which could have arisen from slowed water uptake, slower enzyme kinetics, higher levels of late embryogenesis-abundant proteins, induction of nuclear stress-responsive proteins and imbalanced amount of inhibitor and promoter phytohormones (Achard et al., 2008; Copeland and McDonald, 2012; Abdul Aziz et al., 2021). However, faster germination occurred at a higher temperature than optimal conditions, which could be due to a thermal effect, related to number of heat units required for germination. These results are consistent with previous studies that found stressfully high and low temperatures hindered final germination percentage of five cultivars of canola between 10 to 20 percent (Schwinghamer et al., 2015). Seedling growth was also impeded by both high and low temperatures, resulting in lower fresh weight and seedling vigor indices than optimum conditions (Zhang et al., 2015). Hence, discovery of technologies that improve seed germination and seedling establishment are of importance in agriculture. Here, the application of a bacteriocin could enhance canola germination percentage, radical and shoot length, seedling fresh weight and seed vigor compared to the controls under both low and high temperature stress conditions. To be more precise, among three levels of the compound, Thu2 considerably ameliorated negative effects of stress and showed high stimulatory impacts on both seeds and seedlings. This result is in line with findings of Gautam et al. (2016), which highlighted the response of soybean to 10^-9^ M and 10^-11^ M Th17 during germination and seedling development under low temperature. A similar conclusion was reached by He (2009) for Th17 treated corn seeds under a combination of stresses, including low temperature, salinity and drought stress under both controlled environment conditions and a cool spring climate in the field. In another study, the effect of LCO, a compound produced by *B. japonicum* 532C, Th17 and chitopentaose were tested on high oil content cultivars of canola. Treatment with 10 ^-6^ M LCO accelerated germination at 10 °C while seeds treated with 10^-9^ M Th17 had more even and uniform germination (Schwinghamer et al., 2015). LCO has been shown to be a stimulant for seed germination and seedling establishment as illustrated in a large body of published research, whereas Th17 has not been well studied (Kidaj et al., 2012; Suganya et al., 2016; Nandhini et al., 2018). The underlying mechanism of LCO and Th17 in soybean germination under stressful conditions was studied through proteomic approaches. The results indicated that energy, carbon and nitrogen metabolic pathways were influenced by signal compounds under stressful environments. Some notable stress-related proteins, including PEP carboxylase, rubisco oxygenase large subunit, pyruvate kinase and other proteins are up regulated in response to LCO and Th17 treatments under stressful conditions (Subramanian et al., 2016). Similar results were observed where over 600 genes, mostly related to stress and defense, were up regulated in LCO treated soybean leaf tissue under stressful conditions (Wang et al., 2012). These findings highlight that signal molecules can alter metabolism and physiology to assist in alleviating sub-optimal conditions and improve germination and early growth.

### Th17 effects on canola aboveground growth

Unfavourable temperature, as a major environmental factor, considerably affects plant growth and development. Some plants adapt themselves by altering their morphology, while others adjust their physiology or exhibit changes in gene expression, which modifies how they grow, survive and tolerate stresses (Raza et al., 2020; Gupta et al., 2020). However, susceptibility or tolerance is directly associated with plant lifecycle stage. We hypothesized that during vegetative growth of canola, different levels of Th17 would assist in tolerating stressful temperatures and enhance growth variables. The exposure of canola to cold conditions in northern climates like western Canada is unavoidable in early spring because early spring sowing is required to ensure that the crop matures without suffering yield losses due to summer heat stress. Canola, as a cold-acclimated crop, has the capacity to develop cold stress tolerance, known as cold acclimation, once exposed to chilling but not freezing temperature, although its growth might be slowed. Furthermore, the likelihood of high temperature occurrence during mid to end of canola vegetative growth is relatively high, as we experienced during May-June of the 2020 and 2021 canola field seasons in eastern Canada (unpublished data). In this study, plant height, leaf area and biomass accumulations were negatively altered, in contrast to optimal temperature, after 3 weeks of growing in the 10/5°C temperature range, but surprisingly application of the signal molecule efficiently acted as an alleviator and effective plant growth regulator. In contrast, canola showed positive responses to 30/25 °C temperature, suggesting acclimation ability to moderately high temperature. This could arise from thermomorphogensis, a group of morphological and architectural alterations caused by higher ambient temperatures, below the heat-stress level (Quint et al., 2016; Casal and Balasubramanian, 2019; Ludwig et al., 2021). Based on this, canola height was significantly enhanced across all treatments at moderately high temperature, which may indicate that the plant shifted from vegetative stage to flowering by stem elongation; leaf area and fresh biomass were also increased while irrigation with Thu2 and Thu3 resulted in higher levels of these variables than the control and Thu1 treatments. In contrast to many previous studies, which indicated heat as a growth inhibitor, in our experiment canola growth and development continued well at higher temperature, implying that heat acclimation ability, with assistance of the signal compound, could be quite high. However, stress-induction stage, duration of stress and combination with other stressors play a significant role in response to heat. A body of research has taken shaped around the effects of high temperature on crops, but only a few studies have examined the interaction of microbial signal molecules and high temperature on plants (Kilasi et al., 2018; Hussain et al., 2019; Anwar and Kim, 2020; Ayub et al., 2021; Lohani et al., 2021; Ahmad et al., 2021). Our findings fit well with previous studies that reported the interaction of 10^-6^ M LCO irrigation with relatively high temperature (30/30 °C), resulting in 1 more leaf per canola plant, as apposed to the 25/20 °C water-treated controls, which were unifoliate. Similarly, Th17 treatment increased the dry biomass of canola cultivar 04C111, by approximately 70 mg under saline (0.1 M NaCl) and moderately high (30/30 °C) temperature conditions (Schwinghamer et al., 2016). In this study, we observed that two concentrations, Thu2 and Thu3, had considerable positive impacts whereas the higher concentration level, Thu1, showed no significant stimulatory effect compared to the control. It is not surprising that such results were reported because most of the microbially produced substances are signalling molecules which are similar to phytohormones in that their presence, at very low concentrations, has long been recognised to either promote or inhibit plant growth (Backer et al., 2018; Antar et al., 2021). More than this, there are some other factors that highlight the effect of concentrations, including plant species, stage of life, growth conditions, method of application and so on. While certain chemicals can significantly improve plant growth at extremely low concentrations, others must be used at relatively high levels to have a positive impact.

### Effects of Th17 on root architecture

Root growth relies on cell production within the root meristem, cell expansion and differentiation in which environmental factors play a vital role as stimuli. The root system possesses spatial architecture by which it can dynamically adapt itself to changes in the environment, such as shifts in temperature (Bardgett et al., 2014; Onwuka and Mang, 2018). It is worth noting that unlike the broad optimum temperature ranges for the development of aerial parts of various species, optimum root temperature (12-20 °C) is quite similar for almost all species except for tundra plants (Bell and Bliss, 1978). When plants are exposed to low and high temperatures, changes in the root system occur (Alsajri et al., 2019; Fonseca de Lima et al., 2021). In this study, root system growth was inhibited by low temperature, which is in line with previous studies (Rymen et al., 2007; Shibasaki et al., 2009; Zhou et al., 2021). This could be explained by the fact that low temperature hinders cell cycle progression and cell division in root meristems. In addition, lower carbohydrate assimilation under chilling conditions impedes nutrient allocation to the underground system. Interestingly, the interaction of temperature and Th17 was significant; root system architecture was positively modified by irrigation with the compound, particularly Thu3-treated roots, which produced the highest length, surface area, volume, and dry weight across all temperatures. This might be associated with the role of Th17 in phytohormones modifications. In this regard, Prudent et al. (2015) reported that abscisic acid (ABA) was the first plant hormone to be considerably increased in soybean leaves and roots after the application of Th17. This phytohormone facilitates root elongation by restricting ethylene production, suggesting that a reason for changes in root architecture could be a shift in the balance of plant hormones (originating at the roots). As such, Th17 treatment could cause plants to modify their ABA content, which may have resulted in maximal root growth. However, to ascertain the mode of action of Th17 in canola roots, precise phytohormone measurements would be required in future studies.

### Th17 and physiology characteristics

Most physiological functions of plants, including photosynthesis and transpiration are impacted by temperature. Stomatal conductance controls both carbon assimilation, and transpiration, and they mutually influence one another. Hence, temperature affects stomata indirectly by alterations in plant water status and vapor pressure deficit. We determined that photosynthesis increased linearly with time and was maximum under optimal temperature conditions whereas a considerable decline was seen across all physiology variables at low temperature. In accordance with our results, many studies have demonstrated a significant reduction of carbon assimilation rate and stomatal conductance at low temperatures, which is partly explained by disruption of chloroplast structure, thylakoid membrane damage, reductions in enzyme activities, decreased chlorophyll levels, reductions in expression and resulting concentrations of photosynthesis proteins, and reduced electron transport (Liu et al., 2012; Paredes and Quiles, 2015; Li et al., 2018; Khan et al., 2019; Zhao et al., 2020). Regarding high temperature, in line with many studies, carbon assimilation rate decreased with increasing temperature although in our study, the Th17 application could mitigate the reduction. In this regard, Thu2 and Thu3 enhanced photosynthesis rate compared to the control. Increasing conductivity with time under optimal conditions, coupled with more diffusion of CO_2_, resulting in greater assimilation rates, whereas it caused higher transpiration rates which could lead to reduced leaf water potential at high temperatures. These results agree with those studies that reported stomata opening and increased transpiration at higher temperatures (Lu et al., 2000; Mott and Peak, 2010; Xu et al., 2016; Urban et al., 2017), while our findings are in contrast with some experiments that observed no significant changes of conductivity and/or closure of stomata (von Caemmerer and Evans, 2015; Abdulmajeed et al., 2017; Hlaváčová et al., 2018). It is worth noting that higher leaf temperature causes reduction in protein translation, chlorophyll breakdown, rubisco inactivation, photosystem II activity, electron transport chain activity and increased damage to photosynthetic apparatus (Kang et al., 2017; Wang et al., 2018a; Balfagón et al., 2019; Hu et al., 2020). As such, lowering leaf temperature by transpiration would be an approach to compensate for assimilation reduction which could be beneficial, even if, to some extent, water loss was not constrained. We observed greater transpiration rates in Th2 and Th3 treated plants which meant enhanced photosynthesis came at the cost of losing more water; however, it would assist in cooling leaves when water is not a limiting factor; therefore, photosynthesis systems could function more efficiently. This might be one of the explanations for continued increase of photosynthesis in Thu2 and Thu3 plants despite lower intercellular CO_2_ amounts than the control. Lee et al. (2009) demonstrated that application of 10^-9^ and 10^-11^ M Th17, either through root irrigation or leaf spray enhanced soybean photosynthetic rate and leaf greenness. Our finding partly ties with a conclusion reached by Prudent et al. (2015); they demonstrated the positive effects of Th17 treatment on improving carbon assimilation in water-stressed soybean plants, but this was associated with an increased level of ABA, resulting in stomatal closure and reduced transpiration which is opposite to our findings.

## Conclusions

Our results demonstrated that the Thu2 solution interacted with stressful temperatures to affect canola germination and seedling development. Notably, germination time was significantly reduced when seeds were treated with Thu2 under low temperature conditions, which would be a solution for slow germination of spring-sown canola. The moderately high temperature along with Thu2 and Thu3 treatments could produce taller plants with more leaf area and fresh biomass accumulation than control plants grown under optimal conditions. Watering plants with the bacterial compound promoted root growth and development, including root length, root volume and root diameter, which could facilitate the uptake of water and nutrients. Interestingly, two levels of Th17 had stimulatory effects on photosynthetic rate although the mechanism underlying this is not yet clear. Considering these findings, it seems likely that this signal molecule had stimulatory effects, particularly under stressful temperatures, but we remain inconclusive regarding its mode of action.

## Data availability statement

The original contributions presented in the study are included in the article/supplementary material. Further inquiries can be directed to the corresponding author.

## Author contributions

MN designed the experiment, collected data and wrote the manuscript. IY assisted in data analysis. DS advised on scientific approach and provided editorial input and intellectual context as well as funding. All authors contributed to the article and approved the submitted version.
